# Characterization of AAV vectors: A review of analytical techniques and critical quality attributes

**DOI:** 10.1016/j.omtm.2024.101309

**Published:** 2024-07-30

**Authors:** Theodoros Kontogiannis, Julian Braybrook, Christopher McElroy, Carole Foy, Alexandra S. Whale, Milena Quaglia, C Mark Smales

**Affiliations:** 1 School of Biosciences, Division of Natural Sciences, University of Kent, Canterbury, Kent CT2 7NJ, UK; 2 National Measurement Laboratory at LGC, Teddington, Middlesex TW11 0LY, UK; 3 Reading Scientific Services Ltd, Reading Science Centre, Whiteknights Campus, Pepper Lane, Reading Berkshire RG6 6LA, UK; 4 National Institute for Bioprocessing Research and Training, Blackrock, Co, Foster Avenue, A94 X099 Mount Merrion, Dublin, Ireland

**Keywords:** AAV, viral vector, critical quality attributes, CQAs, gene therapy, analytical methodologies, characterization, capsid identity, genetic identity

## Abstract

Standardized evaluation of adeno-associated virus (AAV) vector products for biotherapeutic application is essential to ensure the safety and efficacy of gene therapies. This includes analyzing the critical quality attributes of the product. However, many of the current analytical techniques used to assess these attributes have limitations, including low throughput, large sample requirements, poorly understood measurement variability, and lack of comparability between methods. To address these challenges, it is essential to establish higher-order reference methods that can be used for comparability measurements, optimization of current assays, and development of reference materials. Highly precise methods are necessary for measuring the empty/partial/full capsid ratios and the titer of AAV vectors. Additionally, it is important to develop methods for the measurement of less-established critical quality attributes, including post-translational modifications, capsid stoichiometry, and methylation profiles. By doing so, we can gain a better understanding of the influence of these attributes on the quality of the product. Moreover, quantification of impurities, such as host-cell proteins and DNA contaminants, is crucial for obtaining regulatory approval. The development and application of refined methodologies will be essential to thoroughly characterize AAV vectors by informing process development and facilitating the generation of reference materials for assay validation and calibration.

## Graphical abstract

**Figure:**
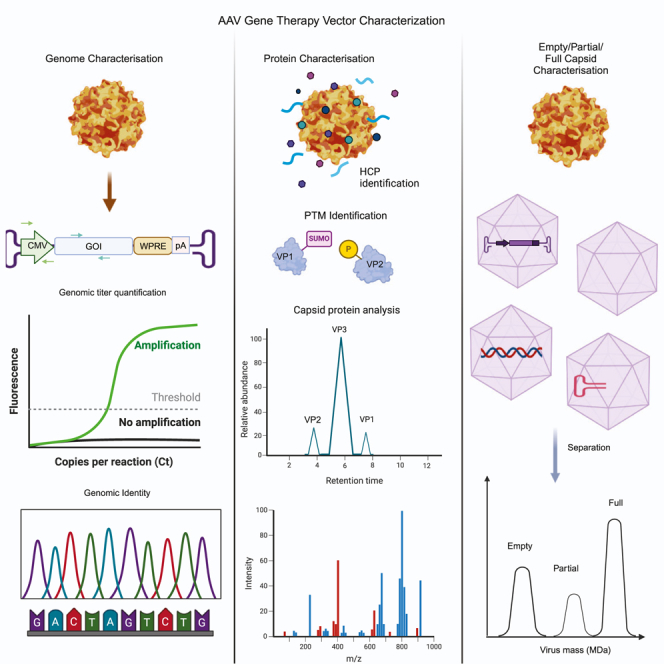

## Teaser

Kontogiannis and colleagues suggest that advanced analytical techniques are necessary for evaluating the critical quality attributes of viral vectors. Due to the variability between AAV serotypes and production batches, the development of reference materials and standardization of the characterization workflow are essential for enabling consistency and comparability between studies.

## Introduction

For gene therapy, viral vectors are commonly used to deliver therapeutic genes into patients' target cells with the aim of treating genetic diseases. To ensure that a viral-vector drug product is safe for testing in clinical settings and for release to the market, it is essential that the final product complies with the predetermined specifications and regulatory requirements of the appropriate regulatory agencies. The closer the product is to market release, the stricter these specifications are.^1^ Validated and precise analytical assays are used to determine whether the product complies with the set specifications by demonstrating that the critical quality attributes (CQAs) are within the predetermined range.^2^ CQAs include identity, purity, potency, safety, and stability of viral vectors, and their accurate characterization is mandated by regulatory agencies such as the US Food and Drug Administration (FDA).^3^ Furthermore, understanding how CQAs affect the product’s efficacy and safety is critical for streamlined gene therapy development and these attributes should be consistent from batch to batch. However, the biomanufacturing of viral vectors is a complex process and small changes in the vector design, such as choosing a different promoter or transgene, selection of a different production platform or upstream bioprocessing conditions (e.g., medium, culture temperature), or use of a different purification technique, can lead to the production of viral vectors with different characteristics that could substantially influence their CQAs, infectivity, and safety.^1^ The monitoring and characterization of viral vectors is thus an important step in process development.

Despite the importance of analytical analysis of viral vectors, current assays, and reference materials used for their characterization are much less well defined than for biotherapeutic proteins. For example, the National Institute of Standards and Technology (NIST) monoclonal antibody reference material 8671 underwent extensive multi-laboratory studies to characterize its physicochemical and immunochemical properties, purity, concentration, biological activity, glycan occupancy, and post-translational modifications. Similar evaluations are needed for viral vectors, but their complex composition and variable properties require additional assays to assess attributes such as particle occupancy, infectivity, genome integrity, and stoichiometry. Additionally, unlike monoclonal antibodies, viral-vector manufacturing and downstream processing lack industry-wide standardization, contributing to variability and challenges in ensuring consistent quality and efficacy.^2^^,^^4^^,^^5^^,^^6^ Optimizing currently utilized assays or developing new high-throughput and reproducible analytical techniques would help the scientific community understand how CQAs can impact the efficiency and safety of the final product.^6^ Equally, to ensure rapid implementation of such methods, translational research and higher-order methods are required. Here, we offer a comprehensive overview of how metrology can contribute to the measurement of the CQAs of viral vectors and present up-to-date data on the characterization of adeno-associated virus (AAV) vectors manufactured as advanced biotherapeutics using current state-of-the-art analytical techniques.

## Metrology in biotherapeutic viral vector characterization

Metrology is defined as the science of measurement, and as such it is a critical aspect of characterizing medicines, including biologics generally, and, for the purposes of this review, specifically biotherapeutic viral-vector batches. Metrology involves using reliable reference materials and/or measurement procedures, adhering to standardized sample preparation and data processing workflows, employing validated data analysis software, and maintaining consistent instrument settings.

Developing a well-defined “measurement framework” for viral-vector characterization is essential to ensure the accurate and consistent assessment of the CQAs of such vectors and underpins the implementation of novel analytical platforms as part of the manufacturing process and of final drug substance and drug product by enabling comparability studies. Although methods have, and continue to be, developed for the characterization of these biotherapeutic modalities, such a framework that is utilized across the industry is yet to emerge.

### Viral vectors and measurement challenges

AAV vectors are among the most widely used vectors in gene therapy.^7^ Characterizing viral vectors is challenging due to their complex nature. AAVs are non-enveloped, with a single-stranded DNA (ssDNA) genome encased within the capsid.^8^ Besides their complex structure, AAVs also exhibit variability among serotypes, as well as between and within batches. This variability includes differences in post-translational modifications (PTMs), stoichiometries of the capsids, and in the sequences of the encapsidated genomes. Therefore, analytical methods capable of characterizing the complex nature of viral vectors are needed.^9^

**Figure 1:**
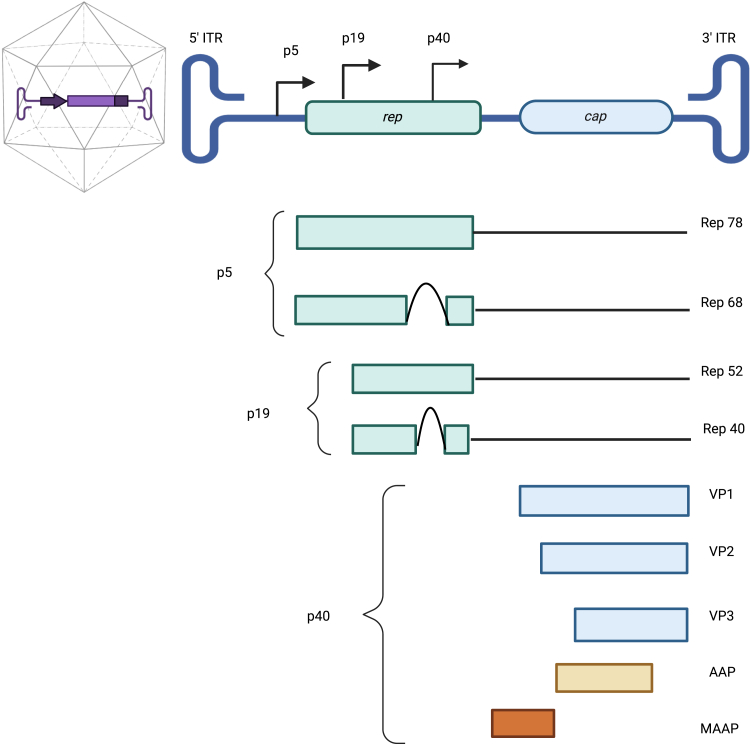
Schematic of the genome organization of wild-type AAV The genome of adeno-associated viruses (AAVs) consists of two main genes, rep and cap, flanked by T-shaped hairpin structures called inverted terminal repeats (ITRs). The rep gene encodes for four non-structural Rep proteins (Rep 78, Rep 68, Rep 52, and Rep 40), while the cap gene encodes three capsid proteins (VP1, VP2, and VP3). Alternative open reading frames (ORFs) of the cap gene encodes for the assembly-activating protein (AAP) and for the membrane-associated accessory protein (MAAP).

This review focuses on the analytical methods used to measure the CQAs of AAV vectors that originate from adeno-associated viruses. The AAVs belong to the Parvoviridae family and are small (∼20 nm) non-pathogenic, non-enveloped, icosahedral viruses that package 4.8-kb ssDNA.^8^^,^^10^ Their genome consists of two main genes, rep and cap, flanked by T-shaped hairpin structures called inverted terminal repeats (ITRs) (Figure 1).^11^^,^^12^

### Viral-vector reference materials

Comparisons of the data from various viral-vector characterization studies are essential for understanding the properties of viral vectors. Incorrect measurements can lead to improper conclusions about the quality of the viral vectors under assessment. In a worst-case scenario, this could lead to the development or release of ineffective or even deleterious treatments. For these reasons, robust metrology science needs to play an essential role in ensuring the accurate characterization and safety of viral vectors destined for use in the clinic.^13^

A key aspect of achieving accurate analytical measurements and reducing the variability of results between different labs is the generation of reference measurement procedures and/or reference materials. However, the generation of reference materials for viral vectors is far from straightforward. The characterization of the components of viral vectors is challenging due to their high complexity, as discussed further below. Moreover, the properties of viral vectors are highly variable and are influenced by process parameters within the upstream and downstream bioprocess. In addition, viral-vector preparations can be sensitive to environmental factors, buffers, and formulation components; therefore, it is important to ensure that their properties have not changed over time. Indeed, changes in the properties of a viral-vector reference material compromises the reliability of the reference material and impacts the supply over time.^14^^,^^15^ Thus, re-evaluating the properties of the reference material periodically is essential to ensure its authenticity. For these reasons, there is currently a limited number and availability of reference materials for different viral vectors used clinically, and there are often limitations on the amounts of a given viral-vector reference material that a laboratory can obtain.

Traceability to the International System of Units (SI) requires the establishment of an unbroken chain of calibrations to reference materials and/or measurement procedures.^16^ Such traceability allows the accuracy of measurements to be established, which is an essential part of quality control.^16^ In reality, achieving traceability to SI is challenging because of the high complexity of viral vectors.^12^ Additionally, whenever changes occur in the manufacturing process, it is essential to demonstrate comparability between different batches. Moreover, standardizing the ancillary raw materials used in viral-vector production is essential in order to guarantee a consistent and reproducible characterization process. This is important because the final product might vary between different manufacturers depending on the intended application, making standardization more challenging. A framework for enhancing the quality and consistency of the ancillary materials has been provided in the documentary standard “ISO 20399:2022 Biotechnology — Ancillary materials present during the production of cellular therapeutic products and gene therapy products.”^17^ Furthermore, “ISO 16921-2: Biotechnology - Gene Delivery Systems - Part 2: Guide for Methods for the Qualification of Viral Vectors” provides guidance for determining the physical and functional titer of viral vectors that should be central in ensuring consistent characterization of viral vectors.^18^

### AAV vector reference materials

As outlined previously, consistency in measurements and protocols is essential in viral-vector research. The lack of standardization in methods used by individual laboratories can make it difficult to compare data across studies. To address this, reference standard materials (RSMs) of recombinant AAV (rAAV) serotype 2 (catalog no. VR-1616) and rAAV serotype 8 (catalog no. VR-1816) have been created to facilitate the comparison of data from preclinical and clinical studies conducted by different laboratories.^19^^,^^20^ The AAV2 and AAV8 reference materials are available through the American Type Culture Collection (ATCC). These reference materials are intended to be used to standardize internal standards and analytical methods, and to enable comparisons of drug product and drug substance batches used in different non-clinical and clinical studies. Furthermore, the reference materials can facilitate the development and implementation of new methods by providing comparability data.

The production and characterization of the ATCC AAV2 (ATCC, ref: VR-1616) and AAV8 (ATCC, ref: VR-1816) reference materials are being overseen by the AAV2 and AAV8 Reference Standard Working Group (AAV2RSWG and AAV8RSWG). The AAV2 and AAV8 reference materials were characterized by 16 laboratories worldwide for the following attributes: (1) the particle (capsid) concentration using enzyme-linked immunosorbent assay (ELISA), (2) vector genome titer of DNase I-treated lysed capsids using quantitative PCR (qPCR), (3) infectious titer using 50% tissue culture infectious dose (TCID50) assays, and (4) purity and capsid identity using sodium dodecyl sulfate polyacrylamide gel electrophoresis (SDS-PAGE). The AAV2 reference material was also characterized for its transduction titer by detecting GFP-expressing cells by fluorescence microscopy.^13^ Table 1 provides an overview of the current methods used for the characterization of viral vectors.

**Table 1:** Summary of analytical techniques used for viral-vector characterization

Method	Scope	Measured CQAs
qPCR^21^	vector genomic titer, infectious titer, detection of DNA contaminants	potency,^a^ purity, safety
dPCR^22^^,^^23^^,^^24^	vector genomic titer, infectious titer, detection of DNA contaminants	potency,^a^ purity, safety
NGS^25^^,^^26^^,^^27^	methylation analysis, identification of packaged sequences and DNA contaminants	identity, purity, safety
ELISA^28^	capsid titer, detection of HCPs	potency, purity, safety
MS^29^^,^^30^^,^^31^^,^^32^^,^^33^^,^^34^^,^^35^^,^^36^^,^^37^^,^^38^^,^^39^^,^^40^^,^^41^^,^^42^^,^^43^^,^^44^^,^^45^^,^^46^	capsid stoichiometry, serotype identification, detection of HCPs and PTMs	identity, purity, safety
OD^47^	vector genomic titer, capsid titer, empty/full ratio	potency, purity
AUC^6^^,^^48^	aggregation, empty/partial/full ratio	purity, stability
TEM^6^	Viral-vector titer, aggregation, empty/partial/full ratio	purity, stability
CDMS^49^^,^^50^^,^^51^	empty/partial/full ratio, capsid identity	identity, purity
AEX^52^	Viral-vector titer, empty/full ratio	potency, purity
SEC-MALS^53^^,^^54^^,^^55^^,^^56^	aggregation, viral-vector titer, empty/full ratio	potency, purity, stability
SLS/DLS, UV-visible^54^^,^^57^	aggregation, viral-vector titer, empty/full ratio	potency, purity, stability
Mass photometry^58^	empty/full ratio	purity

a In the context of nucleic acid measurement, purity refers to presence or absence of nucleic acid contaminants.

## Physical titer

The viral-vector product is evaluated using assays that quantify the physical and infectious titer of the viral vector. Physical titer is a measurement of the total amount of viral-vector particles that are present in the preparation and can be expressed as genome or capsid titer based on the measurement method used.^13^^,^^59^ The genome titer (often expressed as genome copies per milliliter [GC/mL]) is usually compared to capsid titer to estimate the percentage of capsids packaged with the recombinant genome. Empty (or partial) genome capsids are ineffective and thus manufacturing processes attempt to limit or eliminate these, but analytical methods must be sufficient to determine those particles that have no, or partial, genomes within them.

For AAV genome titration, the commonly employed method to quantify vector genomes is by qPCR, and more recently with digital PCR (dPCR), using assays targeting the transgene or elements of the transgene cassette such as the ITRs, woodchuck hepatitis virus posttranscriptional regulatory element (WPRE), promoters, enhancers, and terminators.^59^^,^^60^ However, the limitation of this method is that the design of the vectors may vary depending on the application. Therefore, the same assays often cannot be used for different vector designs. Aurnhammer et al. designed a qPCR method for AAV titration that targeted a 62-nucleotide (nt) sequence of the AAV2 stem region of the ITRs with a limit of detection (LOD) of 50 copies.^61^ In studies evaluating methods for quantifying genomes extracted from purified AAV virions following removal of plasmid DNA, factors such as assay design and primer/probe location were found to significantly influence qPCR and dPCR results, potentially leading to overestimation or underestimation of viral titers (Table 2).^21^^,^^61^^,^^62^ These variable results highlight the need for further work to be undertaken to explore how assay design impacts the resulting titers and whether droplet digital PCR (ddPCR) is a better choice than qPCR for assessing AAV genome titer measurement as it partitions each AAV genome into individual partitions prior to amplification by PCR.^21^ Indeed, with regard to this, Furuta-Hanawa et al. reported that the measurement of AAV titer by dPCR was less affected by the hybridization sites of the primers and probes within the ITRs, compared to qPCR.^22^ Moreover, in a study conducted by Zanker et al., it was shown that further improvement of the accuracy of dPCR can be achieved by using a non-ionic surfactant (Pluronic F68) that reduces plastic adsorption.^23^ However, in the same study, it was also reported that dPCR is impacted by AAV aggregation because it increases the chance of a partition containing more than one genome molecule and thereby leading to titer underestimation.

**Table 2:** Summary of findings from studies investigating the effect of primer/probe location and genome conformation in AAV titration

Study	Materials assessed	Target regions	Methods utilized	Main findings
Aurnhammer et al.^61^	AAV2/8 extracted ssDNA	ITRs EGFP	qPCR	targeting EGFP resulted in 3.6-fold higher genome titers than ITR qPCR
D’Costa et al.^62^	AAV2RSM, AAV8RSM	ITRs SV40 poly(A) GFP	qPCR	i. targeting the ITRs (using the same assay described in^59^) resulted in 6.1 and 5.1-fold higher genome titers for AAV2RSM and AAV8RSM, respectively, than targeting the expression cassette ii. qPCR efficiency is significantly influenced by plasmid standard conformation
Lock et al.^21^	ssAAV (serotypes 2, 2/8,8, 9), scAAV (serotypes 1, 5, 8)	CMV promoter, EGFP, bovine growth hormone, SV40 poly(A) sequences	qPCR dPCR	in scAAVs, dPCR and qPCR demonstrated a ∼2-fold lower titer of CMV compared to EGFP and poly(A). No significant differences were observed between the targets when ssAAVs were analyzed
Furuta-Hanawa et al.^22^	AAV2 RSM, AAV8 RSM	ITRs, SV40 poly(A)	qPCR dPCR	the conformation of the plasmid standard leads to the observation of higher ITR than SV40 poly(A) titers. Instead, dPCR, which does not require a calibration curve for quantification, gave comparable ITR and SV40 poly(A) titers

SV40, simian virus 40; ssAAV, single-stranded AAV; scAAV, self-complementray AAV.

The determination of AAV titer using various methodologies makes it difficult to compare titers between viral-vector lots without comparison to a standard. The use of different techniques, such as qPCR and dPCR, where there are many variations within the two methods (such as different instrumentation, buffers, and analysis software), compound the further variations in sample pre-treatment and therefore can lead to differences in the observed titers. Additionally, the use of different detection chemistries, such as hydrolysis probes or SYBR Green for the same assays, can further compound the differences since the sensitivity and specificity of the two assays are different. Moreover, as mentioned previously, the location of the primers can significantly influence the measured genome titers.^21^^,^^22^^,^^61^^,^^62^ Furthermore, a standard curve is required for qPCR, which reduces the accuracy of the genome titer quantification.^62^^,^^21^ In contrast, dPCR relies on the Poisson distribution for the determination of the genome titer without the need for a standard curve. This approach may enhance the reproducibility of the genome titration.^23^ Reproducibility between laboratories can be further increased by using standardized and well-characterized calibrants (ATCC rAAV2 VR-1616 and rAAV8 VR-1816) and adhering to the International Organization for Standardization (ISO) 20395:2019 standard. This international standard provides guidelines for designing, optimizing, and validating assays; establishing metrological traceability; and determining measurement uncertainty in the quantification of nucleic acids.^24^

An alternative way to measure the physical titer is by quantifying the viral proteins and/or the viral particles present in the sample. The most common method for this purpose is ELISA. However, ELISA often overestimates the physical titer as it usually cannot distinguish between free capsid proteins and viral particles.^63^^,^^64^ Grimm et al. conducted a sandwich ELISA using the A20 antibody, which binds to the capsid proteins of assembled AAV2 particles and not to free capsid proteins.^28^ This method, when combined with genome titer measurements, gives an indication of the ratio of empty to full particles present in samples. Moreover, an optical density (OD) measurement is a simple and fast approach also used to determine the capsid and genome titer of viral vectors. However, it is limited by the purity levels of the sample. This method measures the viral-vector titer and empty to full ratio by measuring the absorbance of nucleic acids and proteins at A260 and A280 nm, respectively. However, if impurities that absorb at these wavelengths are present in the sample, then the accuracy of the method is significantly reduced. These impurities can be free-floating DNA, proteins, or even buffer components that absorb at UV range.^47^ Furthermore, in a study conducted by Sommer et al., measurements of capsid-to-vector genome ratios, conducted by OD, electron microscopy (EM), and ELISA/qPCR were compared. The OD method exhibited a good correlation with EM, with R^2^ value of 0.96, and demonstrated strong agreement with ELISA-qPCR, with R^2^ value of 0.995.^47^

## Infectious titer

Not all viral particles are infectious. The total amount of viral particles capable of transducing cells is called infectious titer. A widely used approach to determine the infectious titer involves measuring transgene expression following infection of a permissive cell line with the AAV vector.^65^ Techniques for measuring transgene expression include, but are not limited to, ELISA for protein quantification, RT-qPCR for mRNA levels, and fluorescence-activated cell sorting (FACS) for fluorescence analysis for fluorescent transgenes.

Furthermore, endpoint assays, for example TCID50, are commonly used to determine the infectious titer.^66^^,^^67^ In these assays, cells (usually HeLa) with integrated rep and cap sequences are infected using serial 10-fold AAV dilutions in the presence of wild-type adenovirus 5 and the vector genome replication is detected using qPCR 48 h after infection. Primers and probes targeting the gene of interest, or elements of the transgene cassette, are most often used to determine whether the virus is replicating in the infected cells. The TCID50 assay gives an estimate of the AAV infectious titer based on the dilutions that give a positive endpoint (positive replicates) and is calculated using the Kaerber method.^68^^,^^69^

Another technique commonly used for determining the infectious titer of AAV is the infectious center assay (ICA). In this method, a host cell line that is susceptible to AAV vector infection is grown in a culture dish. The AAV sample is then added to the cells and the cells incubated to allow for virus infection. Next, the cells are transferred to a membrane, where the presence of fluorescently labeled virus particles, or infectious centers (ICs), are detected. The number of ICs is then used to calculate the number of infectious units (IU) per milliliter of the AAV sample. Therefore, neither the TCID50 assay nor the ICA measure transgene expression and/or protein function. François et al. demonstrated that the ratio of viral genome (VG) to IU for AAV8RSM was 40 times lower when determined using the TCID50 assay compared to the ICA assay.^69^ This suggests that the TCID50 assay may have a higher sensitivity in detecting virus particles, or it may overestimate the quantity of infectious units present. These differences between TCID50 and ICA methodologies, as well as the time-consuming nature of both techniques, highlights the need for alternative consistent and accurate methods for measuring the infectious titer of AAV.

Distinguishing between titer and potency assays is important. While titer assays measure characteristics such as VG copies, capsid concentration, and infectivity, they differ from potency assays, which assess therapeutic efficacy through cell-based evaluations of transgene protein expression and function. However, accurate quantification of VG titer remains essential for dosing analysis of AAV vectors.^59^

## Identity assays

### Genetic identity

Verifying the genetic identity of recombinant viral vectors is important for ensuring high product quality and safety. Identification experiments are particularly necessary in settings where various viral vectors are produced, and serotype identification becomes essential.^70^ The verification of the genetic identity of viral vectors is achieved using nucleic acid analysis techniques such as next-generation sequencing (NGS).^25^^,^^26^ Guerin et al. used the Illumina MiSeq system to determine the genetic identity of packaged sequences.^27^ In this study, it was demonstrated that AAV DNA extracts appeared as a mixture of ssDNA and dsDNA molecules, and therefore a double-strand synthesis step was not necessary in their NGS protocol to achieve good coverage and sequence depth. Guerin et al. proposed that the formation of these molecules originates from the complementary pairing of the positive (+) and negative (−) DNA strands following the DNA extraction process. This NGS VG sequencing protocol was successfully used for serotype verification and determination of the sequences packaged into the capsids.^27^ Tran et al. demonstrated that vector genomes can exist as a mixture of species and the chosen production platform might influence their heterogeneity.^71^ In this study, it was reported that AAVs produced by *Spodoptera frugiperda* (*Sf9*) cells had genomes of 1.5–3 kb that were extremely heterogeneous. Among them, unresolved and truncated genomes were identified. On the contrary, the genomes of rAAVs produced in human embryonic kidney 293 (HEK293) cells did not show the same degree of heterogeneity and existed mainly as single species.^71^

### Genome integrity

The genome integrity of AAV particles is a growing CQA as it is important for ensuring the effectiveness and safety of a gene therapy. AAV particles with partial (truncated) genomes can be ineffective or even pose risks to patients if they express truncated or aberrant proteins. Furthermore, the evaluation of the genome integrity of the AAV sample can help determine the appropriate dose needed to be administered to the patients. This emerging CQA can be evaluated using multiplex dPCR, which employs probe assays with distinct fluorophores that emit signals in different color channels. These probes target various regions of the recombinant AAV genome. When all probe assays yield positive signals, it indicates that the particle likely contains a complete genome. Moreover, the incorporation of multiple probe assays in the multiplex dPCR enhances the accuracy of estimating the presence of a full genome.^22^ Another useful method for assessing the integrity of the AAV genome is Nanopore NGS due to its ability to sequence the entire AAV genome without the need for fragmentation of the AAV genome. Moreover, Nanopore NGS provides detailed insights into identifying truncation hotspots, enhancing our understanding of AAV genome integrity.^25^

### Methylation status

The methylation status of recombinant AAV genomes is an area that has not been thoroughly investigated. In one of the few studies to date, Tóth et al. conducted bisulfite PCR sequencing and showed that methylation levels were negligible in wild-type AAV2 genomes.^72^ Specifically, in packaged AAV genomes, the ratio of methylated cytosines ranged from 0% to 1.7%, with an average of 0.6%. Rumachik et al. reported similar results with whole-genome bisulfite sequencing of rAAV genomes.^73^ In this study, it was shown that rAAV genomes are hypomethylated and the methylation percentage observed was similar between HEK293-produced and Sf9-produced vectors. Interestingly, a study has shown that AAV genome integrated into the host-cell DNA is hypermethylated.^72^ Tóth et al. showed that, in integrated AAV genomes, CpG sites were 20.4%–98.3% methylated, with an average of 76% methylated sites. When the methylation status of AAV was further investigated, the least methylated CpG sites were located at the open reading frames (ORFs) encoding protein X.^72^

Determining the methylation levels and patterns in different AAV serotypes and across different batches, and how these factors affect the quality of gene therapies, would establish whether methylation status should be considered a CQA of AAVs.

## AAV capsid identity and stoichiometry

Capsid protein identity is another critical factor of AAVs that should be characterized thoroughly. Common methods of characterization used for serotype identification are ELISA and western blot. Mass spectrometry (MS) combined with high-performance liquid chromatography (HPLC) or capillary electrophoresis (CE) have emerged recently as higher-order and complementary approaches for serotype identification and capsid composition analysis because of their high reproducibility, accuracy, and robustness. In addition, tandem MS (MS/MS) can be used for peptide mapping and PTM identification in viral-vector analyses.^74^ However, to ensure the reproducibility and comparability of data generated by MS, it is essential to follow standardized sample preparation protocols and maintain consistent data acquisition parameters, including ionization method, mass range, and resolution across different studies.

MS is particularly powerful for serotype identification as it can distinguish between highly homologous AAVs and even detect point mutations.^29^ Identification of AAV serotypes traditionally has required time-consuming sample preparation and long analysis times. To address this, researchers have developed MS approaches for rapid AAV serotype identification and characterization of both natural and modified AAV capsids.^30^^,^^31^ In this regard, differences in the intact masses of VP1, VP2, and VP3 between the AAV serotypes can be used to correctly verify which AAV serotype is present in a sample. Methodologies such as SDS-PAGE cannot efficiently separate the same VP protein of different AAV serotypes (e.g., VP1 from different serotypes) due to the small molecular-weight differences between them (Table S1). Moreover, western blot is a time-consuming technique and the antibodies used are likely to cross-react with the capsid proteins of other AAV serotypes due to their high homology (ranges from 55% to 99% homology based on which serotypes are compared).^32^ In contrast, liquid chromatography (LC)MS is a sensitive and robust method that can rapidly measure the masses of intact capsid proteins and identify the AAV serotype(s) present.^31^ It is essential to accurately measure the masses of all three VPs because the mass of one of the VPs could be indistinguishable between two or more serotypes. Jin et al. performed reverse-phase LC-MS to accurately measure the masses of VP1, VP2, and VP3 of different AAV serotypes (1, 2, 5,7, 9, rh10) and the pattern of the VP1, VP2, and VP3 masses was used to identify each serotype.^33^ Jin et al. distinguished the AAV1 and AAV9 serotypes by comparing the mass measurements of VP2 and VP3. Similarly, to distinguish the serotypes AAV1 and AAV6, the VP1 mass was used, since the VP2 and VP3 masses of the two serotypes are only 2–3 Da different. The difference between the predicted and measured masses for all serotypes did not exceed 5 Da in this study.^33^

Furthermore, an alternative method that can be used for fast identification of the AAV serotype is differential scanning fluorimetry. Each AAV serotype exhibits distinct structural stability and therefore this characteristic enables serotypes to be identified based on their capsid melting temperature (Tm). While differential scanning fluorimetry has effectively been used to determine the Tm of various serotypes, distinguishing between AAV serotypes with closely similar thermostability poses a challenge.^34^

It is generally estimated that the intact rAAV capsid is composed of 60 copies of the VP proteins with a ratio of VP1:VP2:VP3 of 1:1:10.^35^ However, Snijder et al. demonstrated that stoichiometry actually depends on the relative expression levels of each VP protein.^36^ The findings from this study suggest that capsid assembly is stochastic and that there is no absolute constant AAV capsid stoichiometry. Furthermore, Wörner et al. used Orbitrap ultra-high mass range (UHMR) analysis to perform high-resolution native MS on different AAV serotypes (1, 5, 8, and 9) and found that each serotype consists of a heterogeneous population of particles with variable VP stoichiometry.^37^ According to the study’s stochastic assembly model, even the most abundant stoichiometry was present in only a small proportion of all AAV9 capsids (<3%), with a bulk average ratio of 3:10:47.^37^ Interestingly, Wörner et al. demonstrated that the VP expression levels vary between different serotypes and expression platforms.^37^ These findings are in agreement with the earlier study of Urabe et al. that reported that VP1 expression is marginally higher in HEK293 cells compared to insect cells by western blot.^38^ Importantly, Bosma et al. demonstrated that VP stoichiometry impacts infectivity. In this study, vectors with a low VP1:VP2 ratio (<0.5) were associated with reduced potency.^39^ In contrast, vectors with decreased levels of VP3 had increased potency and were more efficient in gene transfer *in vivo*. However, more research into how capsid stoichiometry and VP expression can affect vector infectivity, and whether this can be controlled, is necessary. To aid this work, development of robust analytical approaches to accurately define this CQA is required.

### AAV capsid PTM mapping and identification

Identification of PTMs of viral-vector proteins is an important analytical task that has received much less attention to date than for biotherapeutic proteins and thus requires further investigation for better characterization of gene therapy products. The presence of particular PTMs can affect the interaction of viral vectors with cellular components and therefore impact its infectivity and tropism.^40^ PTMs may also impact stability, propensity to aggregate, interactions with host-cell proteins, and other attributes and are thus a CQA. Monitoring of PTMs and an understanding of these and how they change during bioprocessing can inform process development and help ensure product quality and consistency. For example, oxidation of capsid proteins is an important PTM to monitor during downstream processing because it represents a usual degradation pathway for proteins. High levels of oxidation could lead to loss of activity and stability of the gene therapy product.^40^^,^^41^

The importance of PTMs has also been demonstrated by studies showing that the transduction efficiency of viral vectors can be significantly influenced by post-translational modifications of the AAV capsid.^42^^,^^43^^,^^44^^,^^45^^,^^46^^,^^75^ It has been reported that altering/removing a PTM can significantly impact the infectivity and packaging of the viral vector. In a study by Mary et al., mutation of a glycosylation site of the AAV2 capsid N253Q substantially reduced the packaging efficiency of AAV2 (100 times), while mutation of the SUMOylation site K258Q led to reduced transduction *in vitro* (28%) and negligible expression levels of the transgene *in vivo*.^46^ These studies demonstrate it is essential to establish effective workflows for monitoring PTMs throughout the manufacturing process and investigate how different PTMs may affect the infectivity and safety of the viral-vector product.

Although PTMs may vary between different AAV serotypes, Giles et al. showed that, for one in particular, deamidation, the degree observed was similar between the different AAV serotypes (8, 9, 1, 3B, 4, 5, and 7).^44^ They demonstrated that shelf life and vector age influenced the viral-vector deamidation levels observed. The deamidation levels increased as the “age” of the vector increased. Giles et al. also reported that vector transduction efficiency decreased dramatically (>10 times) after the mutation of asparagine (N) residues into aspartate (D), suggesting that deamidation has a substantial impact on the transduction efficiency. Furthermore, it was demonstrated that vector samples with higher levels of deamidation had lower transduction efficiency. Interestingly, deamidation was also present in AAV9 in a pattern and extent similar to AAV8.^44^

Potential N-linked glycosylation is another important PTM to monitor on AAV vectors as it can affect the tropism, immunogenicity, and intracellular trafficking of the viral vector.^42^ Studies have reported conflicting evidence regarding glycosylation importance in AAV2 and AAV8. Although Murray et al. proposed potential glycosylation sites on the AAV2 capsid, both Fourier transform ion cyclotron resonance MS and a study by Liu et al. failed to detect the presence of any glycosylation.^35^^,^^76^ In contrast, Aloor et al. identified N-glycosylation at N499 in AAV8, along with a coexisting non-glycosylated variant.^42^ Further, Mary et al. employed MALDI-TOF/TOF analysis to reveal multiple potential N-linked glycan combinations on the AAV2 capsid, while no O-glycosylation was observed.^46^

A number of studies have been reported with the aim of identifying PTMs on AAVs due to their potentially important impact on viral-vector quality and safety. A summary of the PTMs identified in different AAV serotypes is presented in Table 3. The discrepancies in PTMs identified among the different studies can be explained by differences in the production and purification processes used by each study.^40^^,^^41^^,^^46^^,^^73^ Liu et al. used wide-pore amide-bonded hydrophilic interaction chromatography (HILIC) and MS to characterize the PTMs of two AAV8 lots that were produced using different processes. It was discovered that one lot had higher levels of oxidation while the other had higher levels of phosphorylation.^31^

**Table 3:** Summary of PTMs found on the capsids of the AAV serotypes (1-rh10)

AAV serotype	Reported PTMs	Reference
AAV2	S149 phosphorylation; N253 Hex(4); HexNAc(2); N518, N551 HexNAcylation; S537 dHex Hex(3) HexNac(7); K258 SUMOylation; K544 ubiquitination	Mary et al.^46^
AAV3	A2 acetylation; Y6 phosphorylation; S149 HexNAcylation	Mary et al.^46^
AAV4	K479 ubiquitination	Mary et al.^46^
AAV5	S1, S193 acetylation; N33, N49, N55, N92, N204, N243, N371, N421, N441, N442, N492, N508, N545, N571, N585, Q432, Q472, Q587, Q603, Q687 deamidation; Q118, Q99, Q587, N55 succinimide; D38, D317 isomerization; M224, M394, M468, M494, M546, M568, M596, M634, M635, M670 oxidation; E543 amidation; R227, R436, R396, R559, K160, K450, K675, M468 methylation; K122, K451, K639 ubiquitination; S185, S186, S221, S251, S484, S680, T222, phosphorylation; N308, T376 HexNAcylation; K312 SUMOylation; N292 N-linked glycan core; N428 dHex Hex(3) HexNAc(6)	Zhang et al.^40^; Guapo et al.^41^; Mary et al.^46^
AAV6	A2 acetylation	Mary et al.^46^
AAV7	A2 acetylation; K61 SUMOylation; T252 phosphorylation; S157, N254, N460 HexNAcylation	Mary et al.^46^
AAV8	M1, A2, K530, K623, K643 acetylation; R171, R450, R487, R490, R535, R696, K61, K530, K623, K643, K709 methylation; Y6, S149, S153, S197, S394, S501, S600, S634, S712, T203, Y396, T493, T495, Y615 phosphorylation; T494, T663 O-GlcNAcylation; N35, N57, N94, N254, N255, N263, N305, N385, N410, N459, N499, N514, N517, N540, N630, N653, T494, deamidation; N521 N-linked glycan core; N499 N-glycosylation	Rathore et al.^74^; Aloor et al.^42^; Mary et al.^46^
AAV9	A2 acetylation; Y52, T450, S454 phosphorylation; K84, K316, K557 SUMOylation; N57 HexNAcylation; T702 dHex(3) HexNAc(3); K105, K650 ubiquitination	Mary et al.^46^
AAVrh10	A2 acetylation; Y90, S149, T252 phosphorylation; S157, T332, S449 HexNAcylation; N306 N-linked glycan core; K333, K652 ubiquitination; K709 SUMOylation	Mary et al.^46^

The choice of production platform and the bioprocessing conditions can therefore influence which PTMs are found on viral vectors. Rumachik et al. compared the PTMs of rAAV8 and rAAV1 produced by transient transfection of HEK293 cells with rAAVs produced by baculovirus infection of *Sf9* insect cells.^73^ Even though the type of PTM present in both AAV8 preparation were the same, more extensive PTMs were found in the capsid proteins of insect-produced rAAV8 compared to HEK293-produced AAV8. Although there were more PTMs present in baculovirus-produced AAV8 and AAV1 vectors than the HEK293 derived vectors, the PTMs identified in the different vector lots had a number of commonalities (Table 4).

**Table 4:** Summary of common PTMs found in both HEK293 cell and *Sf9*-produced rAAV8 and rAAV1 vectors

Modification	PTM position
**PTMs found in both HEK293-produced and *Sf9*-produced rAAV8**^73^	

Methylation	M1
Deamidation	N35, N57, N94, N254, N255, N305, N385, N410, N459, N499, N514, N517, N540, N630, N653
Phosphorylation	S149, S153, Y396

**PTMs found in both HEK293-produced and *Sf9*-produced rAAV1**^73^	

Acetylation	M1, K143
Phosphorylation	S149, S467
Methylation	K61, K459, R465, R485, K528

The identification of PTMs on viral vectors is a relatively new field and few studies have explored this subject. Extensive methods for PTM characterization on proteins have been developed, particularly for biotherapeutic proteins used in the clinic, and there is thus an opportunity to develop and apply such methods to the characterization of AAVs. In particular, MS-based approaches are well suited for high-resolution analysis of PTMs on viral vectors for characterization of products but also to monitor PTMs for process development purposes. The impact of conserved PTMs and how these change between serotypes or different batches could be investigated once the necessary assays are established.

## Analytics and viral vector purity

It is essential to ensure that the final vector product is sufficiently pure and of consistent “makeup” to meet the relevant specifications. For this reason, monitoring the purity during downstream processing development becomes essential. The two main categories of impurities are process-related and product-related impurities.^77^^,^^78^ These can be introduced into the system from the producer cells or from components of the upstream and downstream processing methods. The choice of assay for detecting or monitoring impurities depends on the specific type of impurity. For example, ELISA is traditionally used to quantify the total amount of host-cell proteins (HCPs) present, while the total protein content can be measured using a Bradford protein colorimetric assay with a sensitivity of 10 mg/mL.^64^ Residual HCPs, derived from host cells used to produce the recombinant AAV vector during the manufacturing process, pose a potential challenge in ensuring product purity. More recently, MS has emerged as a technique for identifying co-eluting HCPs and product impurities in the samples of interest. MS offers a significant advantage over ELISA for this purpose as it can identify and measure a substantially wider range of specific HCPs, without the need for specific antibodies for each individual protein. Total DNA content can be measured by spectrofluorimetric methods using DNA-binding fluorescent dyes.^64^ Furthermore, host-cell DNA (HCDNA) is commonly detected by qPCR targeting sequences that are frequent in the host-cell genome (e.g., Alu repeats).^79^

### Analysis of empty, full, and partial capsids

**Figure 2:**
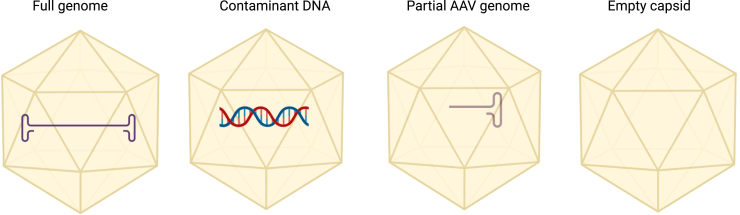
Schematic of the diverse AAV products that can be found in an AAV production batch Product-related impurities can include empty capsids, capsids containing partial genomes, and capsids encapsidating residual DNA from the rAAV production process.

Separation and removal of product-related impurities is a challenge due to their close similarity to the desired product (Figure 2). These impurities must be identified and kept to the lowest possible levels as they are usually at best inactive but can also potentially pose a safety risk, although there are contrasting reports around the impact of such impurities.^80^^,^^81^ For example, Mingozzi et al. reported that including empty capsids in the final formulation of AAV can actually help vector treatment doses overcome humoral immunity in patients receiving gene therapy.^82^ The capsid content can be characterized by a number of methods, including analytical ultracentrifugation (AUC), transmission EM (TEM), anion exchange chromatography (AEX), charge detection MS (CDMS), size exclusion chromatography-multiangle light scattering (SEC-MALS), static, dynamic light scattering (SLS/DLS), qPCR/ELISA, and mass photometry.^6^^,^^40^^,^^53^^,^^58^^,^^83^

#### qPCR/dPCR and ELISA

A widely used method to measure the ratio of full to empty capsids is to divide the number of genome copies detected by dPCR or qPCR by the viral particles measured using ELISA.^6^ The ratio of these two values will give the ratio of full to empty capsids, assuming that one fully intact genome is packaged per viral particle or capsid. However, in addition to the fact that partial capsids and genomes are present in most samples, this approach also has other disadvantages. ELISA is a laborious and generally low-throughput technique. Moreover, as mentioned previously, qPCR requires the preparation of a standard curve, is susceptible to variability in the efficiency of the PCR reaction, and can also be affected by PCR inhibitors, secondary structures, and primer-dimers that may be present in the reaction.^6^^,^^84^ Two-dimensional dPCR can also be employed to distinguish between truncated and full genomes using assays targeting multiple genomic sites. However, a limitation of this approach is that, if two capsids with truncated genomes, each containing one of the target sites, are partitioned into the same droplet, it will result in a false double-positive result.^22^^,^^23^

#### Analytical ultracentrifugation

Sedimentation-velocity AUC is a method that utilizes centrifugal force to separate particles based on their size and molecular weight. The sedimentation profiles are used to determine the distribution of particle sizes and molecular weights. This technique is used to detect the presence of aggregates and separate the empty, partial, and full capsids in a sample. Even though this method has high reproducibility (coefficient of variation [CV] 2%), it requires a large amount of sample for analysis and is a low-throughput method. For these reasons, AUC is commonly used as an orthogonal method to validate other more convenient methods.^6^^,^^48^

#### TEM

TEM can be used to evaluate the size, morphology, and purity of rAAV particles. However, TEM has some limitations that should be considered when using as an analytical approach. For example, errors in measurement can arise from weakly or uneven stained particles and from distortion of samples during preparation. Furthermore, TEM is a low-throughput technique and requires specialized equipment and a lengthy sample preparation process.^6^

#### CDMS

CDMS is a single-particle technique that involves the simultaneous measurement of the mass-to-charge ratio (*m/z*) and the charge of individual ions. This method has been used to analyze empty, partial, and full viral-vector particles.^49^ Although CDMS is a precise method, the technology is still evolving and it is not currently as mature as other methods.^6^^,^^50^ Barnes et al. used CDMS and gel electrophoresis to characterize the genomic content of rAAV8 vectors produced in insect cells by baculovirus infection.^51^ It was shown that a heterogeneous population of rAAV8 vectors was present in the preparation. In addition to empty and full particles, the authors reported the presence of partial capsids packaged with a wide range of different length DNAs, likely due to the packaging of truncated genomes, host-cell DNA, or plasmid sequences. Similarly, Pierson et al. used CDMS to distinguish between full AAV8 capsids and empty or partial capsids, reporting a mass of 3.729 MDa corresponding to empty AAV8 capsids and a second mass at approximately 5 MDa corresponding to the full AAV8 particles.^50^ The observed CDMS results were in close agreement with those of EM, demonstrating the accuracy of this method for purity evaluation. Specifically, EM showed that 43% of the vectors were full, 30% were empty, and 27% were ambiguous or partial.^50^ Similarly, CDMS showed that 42.1% of the total capsids were full, 29.1% were empty, and 28.8% were ambiguous/partial.

#### Size-exclusion chromatography-multiangle light scattering

Here, the sample is first separated by size using size-exclusion chromatography (SEC). The sample fractions are then subjected to multiangle light scattering (MALS) as they pass through the column. The scattered light patterns that are produced can be analyzed to determine the size (molecular weight) and shape of the sample molecules.^85^ SEC-MALS is a powerful technique that can be used to study viral-vector aggregation and quantify empty and full capsids.^53^^,^^54^ This method is characterized by a simple sample-preparation process, high accuracy, and precision.^55^ To ensure the validity of the assay, the use of reference vectors that contain known quantities of full, partial, and empty capsids would be beneficial.^56^

#### SLS and DLS

SLS/DLS provides information regarding the titer, empty/full ratio, and aggregation of viral particles. In general, SLS/DLS are methods used to determine the size and size distribution of particles in a sample. This is achieved by shining light on the sample and measuring the scattered light at various angles. The intensity of the scattered light is used to calculate the size and size distribution of the particles. DLS has a low overall precision and low reproducibility. Specifically, the method has been reported to have a 45% CV for capsid titer measurement and 85% CV for measurement of the aggregate content when using multiangle DLS.^6^^,^^54^^,^^57^

Markova et al. used SEC-static light scattering (SEC-SLS) to determine the percentage of full AAV capsids in samples.^86^ This method determined the percentage of full capsids for AAV2, AAV5, and AAV9. In the study, Markova et al. also distinguished empty from full capsids based on their responses to thermal stress as empty capsids are more prone to thermal destabilization.^86^

#### AEX

AEX utilizes the variations in the electrical charge of particles to distinguish between empty and full capsids.^87^^,^^88^ The charge differences can vary between serotypes and transgene cassettes, making it challenging to use this method in a wide range of applications. Despite this, a previous study using the method reportedly had a low CV (1.1%) and moderate precision of (2%).^52^ Furthermore, in the same study, the method was shown to have an LOD and limit of quantification (LOQ) of 1.6 × 10^10^ and 5 × 10^10^ vg/mL respectively.^52^ However, these parameters may vary depending on the sensitivity of the detector used.^2^

Khatwani et al. developed an AEX-HPLC method with a modular discontinuous gradient for high-throughput in-process quantification of empty and full capsids of different serotypes: AAV1, AAV2, AAV3, AAV6, AAV8, and AAV9.^83^ The method had an excellent correlation (r > 0.99) with AUC and cryogenic-TEM (cryoTEM) for the quantification of empty and full capsids, without being time consuming or requiring large amounts of purified material. Similarly, Dickerson et al. used AEX with an isocratic wash to separate and quantify empty and full AAV capsids by exploiting the small difference between the surface charge of empty and full capsids.^89^

The empty/full ratio may differ between different production platforms and even between production runs. Rumachik et al. used cryoTEM and showed that HEK293-produced AAVs have a higher percentage of full capsids compared to *Sf9*-produced AAVs.^73^ Moreover, Galli et al. demonstrated that AAVs produced in *Saccharomyces cerevisiae* contain mainly truncated genomes.^90^ The sequences present in the truncated genomes were mostly ITRs or regions that flank them.

### Analysis of DNA impurities

To ensure the safety of gene therapies, it is essential to characterize, monitor, and remove any potential DNA impurities from plasmids or host cells.^91^ These impurities could be packaged in capsids and trigger immune responses if administered to patients or potentially result in expression of undesirable elements such as antibiotic selection markers on plasmids.^92^ Guerin et al. demonstrated that sequences around the p5 promoter are preferentially packaged into capsids, potentially due to Rep-mediated recombination events, while Brimble et al. reported that packaged DNA sequences located upstream of the p5 promoter on the AAV rep-cap plasmid are transcriptionally active.^71^^,^^93^^,^^94^ The expression of these sequences could potentially cause harmful effects to the patient. Therefore, it is necessary to develop methods that can monitor these impurities and investigate their effects on the safety of the gene therapies.

Lecomte et al. developed a novel NGS method, called single-strand virus sequencing (SSV-seq), to characterize the DNA content of rAAVs and quantify DNA contaminants present in preparations.^95^ The type of DNA contaminant varied when different purification processes were used. The main DNA contaminant present in CsCl-purified AAV samples was mitochondrial DNA (mtDNA), in particular a triple-stranded structure (D loop) found in the main noncoding region of mtDNA22. To the contrary, the main DNA contaminant found in AVB Sepharose affinity chromatography-purified AAV samples were chromosome 15 sequences. Penaud-Budloo et al. used SSV-seq to analyze AAV preparations and reported that the main DNA contaminant in AAVs produced in insect cells was baculovirus DNA.^96^ Yet another study by Tai et al. used a single-molecule real-time sequencing method called AAV genome population sequencing (GPseq) with self-complementary AAVs (scAAVs) to measure the abundance of partial genomes, reverse-packaged genomes, and packaged host-cell DNA.^97^ It was demonstrated that many of these mistakenly packaged genomes were chimeric with the AAV-ITRs. More recently, Zanker et al. used duplex dPCR targeting the cytomegalovirus (CMV) promoter and the beta-lactamase gene and demonstrated the presence of the beta-lactamase sequence in 1.74% of the tested AAV8RSM particles.^23^ Furthermore, Higashiyama et al. developed and successfully implemented a dPCR method targeting the 18S ribosomal RNA (18S rRNA) for the quantification of residual host-cell DNA.^98^ These methods provide a useful tool for rapid characterization of DNA contaminants present in the AAV samples.

Wang et al. compared three types of AAV impurities for their effect on AAV transduction.^99^ These included syngeneic pseudo-vectors that comprised partial AAV genomes sourced from DNA of complete particles, allogeneic pseudo-vectors that had partial genomes distinct from those of the corresponding full particles, and null pseudo-vectors that lacked any DNA within their capsid structures. Interestingly, injection of mice with full AAV vectors in combination with syngeneic pseudo-vectors led to a 2- to 3-fold higher transgene expression. However, injection of mice with the allogenic pseudo-vectors or the empty particles, together with the full AAV particles, did not influence transduction efficiency. Similar results were obtained *in vitro*. It was suggested that this effect occurs because the DNA sequences of syngeneic vectors serve as primers for duplex DNA formation and therefore facilitate second-strand synthesis.^99^

Despite such studies, the impact of empty and partial capsids on an AAV product’s efficacy and safety is unclear. Determining whether there is a threshold number of full capsids that could transduce cells without being affected by the presence of empty or partial capsids would be interesting. Further studies investigating the effect of frequently identified mis-encapsidated DNA sequences and how their location, inside or outside the capsid, could potentially influence the safety and efficiency of the final product are required.

### Host-cell protein analysis

Depending on the production system used and the purification protocol, the viral vector may co-elute with a wide variety of HCPs.^100^ It may even be possible that these are enclosed within the rAAV capsid. Identifying and quantifying these HCPs is an important task for ensuring the safety of the gene therapy products as they could potentially cause an immune response in the person receiving the therapy.^101^^,^^102^^,^^103^ Characterization of HCPs in biotherapeutic protein products is well established; however this is not necessarily the case for viral-vector preparations.^104^ Interestingly, in a study conducted by Rumachik et al., HEK293-produced AAVs were co-eluted with HCPs that were mostly involved in the binding of nucleic acids and proteins for RNA processing, while *Sf9*-produced AAVs were co-eluted with proteins that have roles in proteolysis/endopeptidase activity.^73^ Dong et al. identified 13 proteins co-eluting with AAV purified by CsCl-gradient ultracentrifugation.^100^ Among them, the researchers identified the protein SET. This protein was identified in several batches regardless of the serotype or the transgene used. Interestingly, SET was only found in fractions that contained full AAV capsids. Furthermore, Aloor et al. identified 28 HCPs in intracellular and secreted AAV8 samples, while the galectin-3-binding protein was identified in both samples.^42^ The same host-cell protein was also identified in the human cell produced AAV8 vectors in the study conducted by Rumachik et al.^73^ The HCPs galectin-3-binding protein, nucleophosmin, and fibulin-1 were found in both studies.^42^^,^^73^ Table 5 details the HCPs that have been identified in more than one study on AAV-associated HCPs. Of these, nucleophosmin and the ssDNA-binding protein (mitochondrial) were identified in both AAV8 and AAV2 samples. Further studies are needed to elucidate the role, if any, of these proteins during rAAV production, whether the amounts of these can be reduced or eliminated, and whether there are any implications for safety and efficacy of AAVs due to the presence of these HCPs.

**Table 5:** HCPs that have been identified in rAAV preparations in more than one studies

Host-cell protein	Function	Serotype	Reference	UniProt accession no.
Galectin-3-binding protein	cell adhesion	AAV8	Rumachik et al.^73^; Aloor et al.^42^	Q08380
Nucleophosmin	ribosome biogenesis, centrosome duplication, protein chaperoning	AAV8, AAV2	Rumachik et al.^73^; Aloor et al.^42^; Dong et al.^100^	P06748
Fibulin-1	cell adhesion and migration	AAV8	Rumachik et al.^73^; Aloor et al.^42^	P23142
Single-stranded DNA-binding protein, mitochondrial	mtDNA replication	AAV8, AAV2	Aloor et al.^42^; Dong et al.^100^	Q04837
Ferritin	iron storage protein	various	Crosson et al.^105^; El Andari and Grimm^106^	Q8TD27

**Figure 3:**
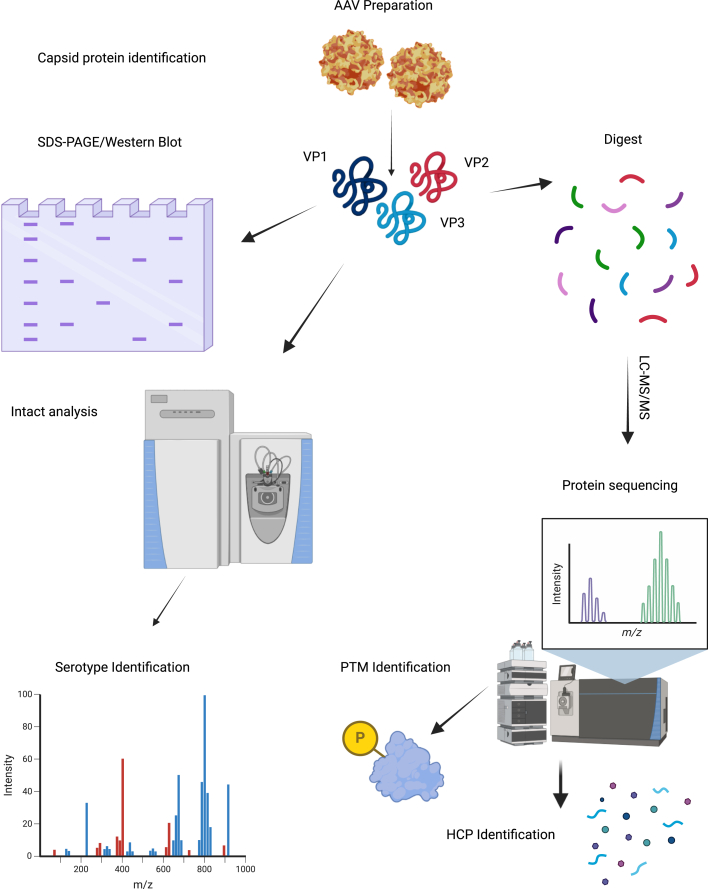
Schematic representation of protein characterization workflows for rAAV preparations Capsid proteins are first identified using SDS-PAGE or western blotting. Serotype identification is accomplished via intact LC-MS analysis, whereas identification of PTMs, HCPs, and protein sequencing is achieved through liquid chromatography-tandem mass spectrometry (LC-MS/MS).

As described above and unlike biotherapeutic protein HCP analysis, where large amounts of work have been undertaken on HCPs and standard ELISA and MS methods exist for monitoring the HCP content, little research has been undertaken to date on HCPs present in viral-vector preparations. As such, there is a need and opportunity to investigate how the HCPs change with different bioprocessing methods and assess the risks that HCPs might pose to the patient.^104^ Furthermore, it remains largely unknown how the location of HCPs may influence the ability to deliver the cargo to cells and achieve transgene expression. HCPs could potentially be located outside or inside the capsid, bind to the genome of the vectors, or be associated with the envelop of enveloped viral vectors. Investigating the impact of the location of HCPs on the safety and effectiveness of the gene therapy product is a research area that needs more attention. In order to facilitate this, the development and application of new methods or approaches that enable the detection and analysis of HCPs in viral-vector preparations is essential. Methods for characterization of the protein content of AAV preparations, including HCP analysis, are depicted in Figure 3.

## Conclusions and future directions

The number of human gene therapies in development continues to rapidly increase. In order to support the design, development, manufacture, and safety of these modalities, the development of more standardized, detailed, and accurate analytical characterization methods of viral vectors is essential. Such assays should be able to define and monitor CQAs of viral vectors to ensure safe and effective therapies. However, this requires complementary analytics that can rapidly and precisely monitor the titer, quality, and efficacy of the viral vectors being produced. Moreover, the regulatory demands for gene therapy products are becoming stricter, with a need for accurate characterization of identity, potency, purity, safety, and stability. To achieve this, high-throughput, precise, fast, and reproducible analytical methods must be developed to ensure safety and efficiency of the product. Further, to ensure comparability of both the quality and quantity across diverse viral-vector lots, it is important to establish harmonization of the entire characterization process. In this regard, reference materials play an essential role in facilitating comparison of results across multiple studies. Additionally, it is important to outline and address potential measurement variability resulting from analytical methods employed in assessing the CQAs of viral vectors. Such comparative analysis will allow the implementation of new and improved analytical methods that advance the measurement, characterization, reproducible manufacturing, safety, and efficacy of viral-vector gene therapies.

## Supplemental information

Document S1. Table S1

Document S2. Article plus supplemental information
