# Supplementary material for: Characterization of AAV vectors: A review of analytical techniques and critical quality attributes
Source: Mol Ther Methods Clin Dev. 2024 Jul 30;32(3):101309. doi: 10.1016/j.omtm.2024.101309 (PMC11372808; doi:10.1016/j.omtm.2024.101309)
Supplement: Document S1. Table S1 [file mmc1.pdf]

OMTM, Volume *32*

Supplemental information

Characterization of AAV vectors: A review of analytical techniques and critical quality attributes

Theodoros Kontogiannis, Julian Braybrook, Christopher McElroy, Carole Foy, Alexandra S. Whale, Milena Quaglia, and C Mark Smales

Table S1. The masses of VP1, VP2, VP3 for AAV serotypes (1-12).

Serotype	Mass VP1 (Da)	Mass VP2 (Da)	Mass VP3 (Da)
AAV1	81,286	66,093	59,517
AAV2	81,856	66,488	59,974
AAV3	81,571	66,319	59,849
AAV4	80,550	65,626	59,529
AAV5	80,336	65,283	59,463
AAV6	81,322	66,096	59,519
AAV7	81,564	66,372	59,101
AAV8	81,667	66,519	59,805
AAV9	81,291	66,210	59,733
AAVrh10	81,455	66,253	59,634
AAV10	81,477	66,271	59,638
AAV11	80,987	65,794	59,696
AAV12	82,106	66,905	59,846

References

Jin, X., Liu, L., Nass, S., O'Riordan, C., Pastor, E., and Zhang, X.K. (2017). Direct liquid chromatography/mass spectrometry analysis for complete characterization of recombinant adeno-associated virus capsid proteins. Hum. Gene Ther. Methods *28*, 255. https://doi.org/10.1089/hgtb.2016.178
